# Drug-resistant tuberculosis can be predicted by Mycobacterial interspersed repetitive unit locus

**DOI:** 10.3389/fmicb.2015.00147

**Published:** 2015-02-24

**Authors:** Wen Yu-feng, Jiang Chao, Cheng Xian-feng

**Affiliations:** ^1^School of Public Health, Wannan Medical CollegeWuhu, China; ^2^Institute of Dermatology, Chinese Academy of Medical Sciences and Peking UnionNanjing, China

**Keywords:** tuberculosis, drug resistance, variable number of tandem repeats, MIRU, Mycobacterial interspersed repetitive unit

## Abstract

It is unknown whether MIRU-VNTR (Mycobacterial Interspersed Repetitive Unit-Variable Number of Tandem Repeat) is associated with drug resistance of *Mycobacterium tuberculosis*. The purpose of this study was to explore the ability of 24 MIRU loci to predict the drug resistance of Isoniazid (INH), Rifampicin (RFP), Streptomycin (SM), Ethambutol (EMB) and Pyrazinamide (PZA). We collected the drug resistance and MIRU loci information of 109 strains of *M. tuberculosis* from an open database. The results of multivariate logistic regression showed that the VNTR polymorphism of MTUB04 was related to INH resistance [odds ratio (*OR*) = 2.82, *P* = 0.00], RFP resistance (*OR* = 1.91, *P* = 0.02), SM resistance (*OR* = 1.98, *P* = 0.01) and EMB resistance (*OR* = 1.95, *P* = 0.03). MIRU40 was associated with INH resistance (*OR* = 2.22, *P* = 0.00). MTUB21 was connected with INH resistance (*OR* = 1.63, *P* = 0.02) and SM resistance (*OR* = 1.69, *P* = 0.01). MIRU26 was correlated with SM resistance (*OR* = 1.52, *P* = 0.04). MIRU39 was associated with EMB resistance (*OR* = 4.07, *P* = 0.02). The prediction power of MIRU loci were 0.84, 0.70, 0.85, and 0.74 respectively for INH (predicted by MTUB04, MIRU20, and MTUB21), RFP (predicted by MTUB04), SM (predicted by MTUB21 and MIRU26) and EMB (MTUB04 and MIRU39) through ROC analysis. Our results showed that MIRU loci were related to anti-tuberculosis drug and could predict the drug resistance of tuberculosis.

## Introduction

According to the Global Tuberculosis Report 2013, 8.6 million people were infected with tuberculosis (TB), and 1.3 million people died from TB in 2012, and there were 3.6% of new cases and 20.0% of retreatment were infected with Multidrug-resistant TB (MDR-TB)^1^. Environment (Lalor et al., 2013), including nutritional status, inadequacy of treatment and gene mutation were the main risk factors of drug-resistant tuberculosis. Among genetic factors, the efflux pump (Da Silva et al., 2011) and gene mutation (Nebenzahl-Guimaraes et al., 2014) have been widely used to explain the formation of anti-tuberculosis drug resistance. A recent study found that there was a correlation between genotypes of tuberculosis strains and drug-resistant tuberculosis (Liu et al., 2012). Mycobacterial interspersed repetitive unit-variable number of tandem repeat (MIRU-VNTR) method is widely used in genotyping the tuberculosis strains. The essence of MIRU (a total of 41, (Supply et al., 2000) in the tuberculosis) was a variable number tandem repeat, of which the number were highly polymorphic.

The exact biological mechanisms underlying the effects of VNTR on mycobacteria remain unclear. Some researchers argued that (Supply et al., 1997) it may play a role in the regulation of gene expression, e.g., the operon of the polycistron having differential gene translation and the structure of the chromosome. Akhtar et al. (2009) reported that when the number of repeat unit of MTUB39 was 4, the expression of the downstream gene *lpdA* was 12 times greater than when the number of was 1. Similar result was also reported in another experiment (Perez-Lago et al., 2013), which found a 1.56-fold difference in *lpdA* when the number of repeat unit of MTUB39 was 4 instead of 3. This research also found that there was no difference in the expression of downstream gene whenever the number of repetition of MIRU10 was 4 or 3. Tantivitayakul et al. (2010) found that there was a lower expression of downstream gene *gfp* when the number of repeat unit of MIRU10 was from 4 to 7 copies. In addition, different tandem repeats may affect the folding of DNA, thereby affecting the attraction, binding and interaction (Olsen et al., 2009) of transcription factor. Existing evidence strongly indicated that MIRU locus may exert a regulation function on the expression of genes nearby. Nowadays MIRU-VNTR method is widely used to analyze the genotyping, epidemic information and other areas of tuberculosis strains. However, it is not yet clear whether MIRU locus is involved in the formation of drug-resistant tuberculosis or not, and its ability to predict the occurrence of drug-resistant tuberculosis is not well unveiled. Hence, in order to find a new way to predict the drug-resistant tuberculosis, we conducted a randomized case-control study (the data was from a published database) to explore the relationship between the MIRU locus and drug-resistant tuberculosis.

## System and methods

109 strains were included in the study, of which 54 strains were from Germany, 20 strains from Ghana, 20 strains from Uganda and 15 strains from former Soviet Union. The inclusion criteria were expressed as follows: First, strains must be *Mycobacterium tuberculosis*. Second, MIRU-VNTR genotyping results of the strains included 24 loci information. Third, there were drug resistance information about Isoniazid (INH), Rifampicin (RFP), Streptomycin(SM), and Ethambutol(EMB). Mycobacterium bovis, Mycobacterium africanum and other non-tuberculosis mycobacteria were not included in this study. Strains of which the susceptibility test was positive were enrolled in the case group, while the sensitive strains were included in the control group.

24 loci and drug resistance data were derived from an online reference database of MIRU-VNTRplus website (http://www.miru-vntrplus.org) (Allix-Beguec et al., 2008). The data in the reference database were provided by the Pasteur Institute in France and the German National Reference Center for Mycobacteria. Technical methods for detecting MIRU loci repetition could be referred to the technical manual supplied by the Pasteur Institute^2^.

Allelic diversity *h* was used to measure the MIRU locus polymorphism. Univariate and non-conditional multivariate Logistic regression analysis were applied in MIRU locus prediction model. The dependent variable was drug resistance result, 1 standing for resistance, 0 standing for sensitivity. Independent variable was the number of repetitions of each MIRU locus (continuous variables). The ROC analysis was used to evaluate the effect of MIRU locus model which may predict drug-resistant tuberculosis. Maximum Youden Index was used to determine the predictor of model. Functional genes near the MIRU locus were analyzed by artemis software. According to the upstream and downstream primers of the study locus, the position of MIRU locus in the genome of standard strains of *M. tuberculosis* H37Rv was determined. Moreover, the functions of the upstream and downstream genes of MIRU loci were analyzed.

## Results

### Distribution of the VNTR, polymorphism and drug resistance of MIRU loci

The number of the repeat unit of 24 MIRU loci ranged from 0 to 13. The minimum h value of MIRU27 was 0.10 while QUB 26 was 0.81. Drug resistance rate that corresponded to different copies of each MIRU locus ranged from 0 to 100% (Table in Supplementary information).

### Univariate analysis of factors of anti-tuberculosis drug resistance

Through the univariate logistic regression analysis revealed a significant relationship between loci and anti-tuberculosis drug resistance. Specifically, for INH resistance, there were MTUB04, MIRU40, MIRU16, MTUB21, MTUB21, MTUB30, MIRU26, MIRU31, MTUB39, OUB26, and MIRU39. For RFP resistance, two loci were MTUB04 and OUB26. For SM resistance, there were MTUB04, MIRU16, MTUB21, MIRU26, MIRU31, MTUB39, OUB26, MIRU39. For EMB resistance, there were MTUB04, OUB26 and MIRU39 (Table in Supplementary information).

### Multivariate analysis of factors of anti-tuberculosis drug resistance

Loci which were statistically significant in Table 2 were set up as the independent variables. The multivariate logistic regression suggested that loci which were significantly associated with anti-tuberculosis drug resistance were as follows: for INH resistance model, there were MTUB04, MIRU40, and MTUB21; for RFP resistance model, there was MTUB04; for SM resistance model, there were MTUB04, MTUB21, and MIRU26; for EMB resistance model there were MTUB04 and MIRU39. In conclusion, there were 5 risk MIRU locus of drug resistance (Table 1).

**Table 1:** **Multivariate analysis of influencing factors of four anti-tuberculosis drug resistances**.

**Model**	**Loci**	**B**	**S.E**.	**Waldχ^2^**	**P**	**OR**	**OR 95% CI**	
							**Lower**	**Upper**
INH	MTUB04	1.04	0.27	14.48	0.00	2.82	1.65	4.81
	MIRU40	0.80	0.24	10.81	0.00	2.22	1.38	3.58
	MTUB21	0.49	0.21	5.52	0.02	1.63	1.09	2.46
	Constant	−8.83	1.83	23.38	0.00	0.00		
RFP	MTUB04	0.65	0.28	5.54	0.02	1.91	1.11	3.28
	Constant	−3.97	0.94	17.94	0.00	0.02		
SM	MTUB04	0.68	0.27	6.62	0.01	1.98	1.18	3.32
	MTUB21	0.53	0.20	6.63	0.01	1.69	1.13	2.52
	MIRU26	0.42	0.20	4.23	0.04	1.52	1.02	2.27
	Constant	−7.01	1.32	28.15	0.00	0.00		
EMB	MTUB04	0.67	0.311	4.60	0.03	1.95	1.06	3.59
	MIRU39	1.40	0.59	5.70	0.02	4.07	1.29	12.89
	Constant	−7.64	1.95	15.42	0.00	0.00		

### ROC analysis of four drug resistance

The areas under the ROC curve (AUC) of INH, RFP, SM, and EMB were 0.84, 0.70, 0.85, and 0.75. The predictive values of prediction models of drug resistance ranged from 0.09 to 0.26, and the Youden Index ranged from 0.40 to 0.61 (Table 2).

**Table 2:** **ROC analysis of four drug resistance**.

**Model**	**AUC**	**S.E**.	**P**	**AUC95%CI**	**Positive if greater than or equal toa**	**Sensitivity**	**1-Specificity**	**Yueden index**
INH	0.84	0.05	0.00	0.74–0.93	0.24	0.83	0.22	0.61
RFP	0.70	0.09	0.02	0.54–0.87	0.09	0.75	0.35	0.40
SM	0.85	0.04	0.00	0.77–0.94	0.13	0.92	0.32	0.60
EMB	0.75	0.08	0.01	0.59–0.92	0.26	0.54	0.08	0.46

### Bioinformatics analysis of each MIRU locus

We search each MIRU locus and relative positions of functional genes adjacent to five MIRU loci from the genbank databases, founding that MTUB04 adjacent to *hspR* gene, MTUB21 adjacent to *Rv1729c* gene, MIRU40 adjacent to *rplB* gene, MIRU26 *Rv2680* and MIRU39 adjacent to *eccCb1* gene. The detailed information of each MIRU locus and relative positions of functional genes adjacent to five MIRU loci were listed (Table 3).

**Table 3:** **Bioinformatics analysis of relative gene adjacent to each MIRU locus**.

**Loci**	**Unit length**	**Repeat**	**Adjacent gene**	**Relative position**
MTUB04	51	2	*hspR*	8 bp duplication
MTUB21	33	2	*Rv1729c*	−13 bp
MIRU40	54	1	*rplB*	−46 bp
MIRU26	51	3	*Rv2680*	51 bp duplication
MIRU39	47	2	*eccCb1*	−3 bp

## Discussion

Our study showed that anti-tuberculosis drug resistance (INH, RFP, SM, and EMB) were well correlated with MTUB04, MTUB21, MIRU26, MIRU39, and MIRU40. It revealed that the drug resistance could be predicted by these loci. The prediction power of MIRU loci were 0.84, 0.70, 0.85, and 0.74 respectively for INH (predicted by MTUB04, MIRU20, and MTUB21), RFP (predicted by MTUB04), SM (predicted by MTUB21 and MIRU26) and EMB (MTUB04 and MIRU39) through ROC analysis. Except for the poor sensitivity of resistance prediction model for EMB, the accuracy and sensitivity for other four models were high. Especially, MTUB04 locus had the ability to predict all four kinds of drug resistance, and MTUB21 locus could predict both INH and SM resistance.

Because report about the specific function of MIRU loci was not found, we suspected that some gene next to these loci may be paying an unknown role. For MTUB04, gene *hspR* was located at its upstream and there was 8 bp overlapping between them. HspR, binding to the HAIR (three HspR associated inverted repeats) sequence in the upstream of the *dnaK-hspR* operon, was a repressor of DnaK's (heat shock protein HSP70) (Narberhaus, 1999) expression, and the activity of HspR was regulated by the molecular chaperones DnaK (Bucca et al., 2000). Previous studies showed that part sequence in the 3′ terminal of gene coding strand knock-out HspR almost had no binding activity with DnaK (Bandyopadhyay et al., 2012), then, causing the binding activity of HspR to HAIR reduced, and at last resulting in overexpression of DnaK. Xin et al. found (Xin et al., 2013) that in A549 cells, the overexpression of HSP70 could up-regulate the expression of P-gp transporter protein at transcriptional level. Meanwhile, INH, RFP, and EMB were the substrates of P-gp (Rakash et al., 2003; Hartkoorn et al., 2007; Pin-Fei, 2010), and the upregulation of P-gp could reduce drug concentration of INH, RFP, and EMB in mouse tissues (Rakash et al., 2003; Pin-Fei, 2010). Some studies have found that repeated sequences of *ALU* could inhibit the expression of the reporter gene *GFP* in the upstream (Li et al., 2012). Therefore, we hypothesized that increasing the number of repetitions unit of MTUB04 locus can inhibit the expression of HspR or the sequence of the C' terminal of HspR, thus leading to the occurrence of drug resistance.

The gene *Rv1729c* in the downstream of MTUB21 locus, could express S-adenosine methionine-dependent methyltransferases^3^. Strong evidences showed that the knockout of *mmaA4* or *hma* encoding SAM-MTs proteins(Cole et al., 1998) may inhibit the synthesis of two kinds of Mycobacterium mycolic acid, Keto meromycolic and Oxygenate methyl meromycolic (Dubnau et al., 2000). Some studies showed that clinical efficacy of patients infected with isoniazid and rifampicin resistant *M. tuberculosis* strains could be improved with the use of delamanid inhibiting the synthesis of the two kinds of mycolic acid mentioned above (Gler et al., 2012). One of the mechanisms underlying for INH resistance is that INH oxidized by peroxidase attacks mycolic acid, the main constituent of the cell membrane of *M. tuberculosis*, resulting in the death of mycolic acid. The possible mechanism is that MTUB21 may exist as an enhancer to increase the transcription of *Rv1729c*, resulting in the overexpression of mycolic acid, thus, forming the INH resistance. For SM resistance, the overexpression of mycolic acid may enhance the permeability barrier function of the cell wall, and then prevent penetration by SM.

The gene *Rv2680* located in the downstream of MIRU26, the expression product of it may be the enoyl coenzyme A hydratase^4^. Enoyl-CoA hydratase is a key enzyme for the synthesis of *M. tuberculosis* meromycolic(Strong et al., 2003), involved in the oxidation of β fatty acids. MIRU26 locus may serve as an enhancer to increase the overexpression of enoyl-CoA hydratase, and the penetration of the barrier function of the cell wall, thus resulting in the occurrence of SM resistance.

Existing studies found that when the number of repeat unit of MIRU39 locus was 4, the gene *eccCb1* in the downstream had a higher transcription level (Refaya et al., 2012). The expression product of eccCb1 were ATP enzyme family members, constituting the EXS-1 transport channel together with Rv3870 for the transport of a variety of substrates (Champion et al., 2009) such as EspR (Rv3849), EspA (Rv3616c), EspB (Rv3881c), EspC (Rv3615c), ESAT-6, and CFP-10. We hypothesized that with the increasing number of the repeat unit of MIRU39 locus, the efficiency of the EXS-1 transport channel was increased, which then increased the efflux of EMB, and finally resulted in EMB resistance. However, whether EXS-1 transport channel has effect on the substrate EMB is still an open question.

Gene *rplB* which was in the downstream of MIRU40 locus may express 50S ribosomal protein L2^5^. Reports showed that it participated in the 50S subunit's connection to 30S ribosomal subunit, binded site of tRNA, contacting with16sRNA partly^6^. However, the question that by what mechanisms can rplB allow *M. tuberculosis* to become resistant to INH still can not be explained.

Overall, our findings confirmed that some of the MIRU loci can predict the drug-resistant tuberculosis. The possible mechanism involved was that MIRU locus could increase or decrease the transcription of functional genes nearby, resulting in the up-regulation of the expression of efflux pump, P-gp, meromycolic, mycolic acid and a plurality of other components. MIRU-VNTR was selected as the first choice for genotyping of *M. tuberculosis* by Centers for Disease Control and Prevention of U.S., due to simple operation and low cost (Cowan et al., 2012). Therefore, this study would not only enrich the knowledge of drug resistance of tuberculosis but also provide the probability of further exploration of the mechanism of anti-tuberculosis drug resistance. Meanwhile, it would be a potential cost-effective way to predict drug-resistant tuberculosis through MIRU loci method. However, the expression level of upstream and downstream genes of MIRU loci were not tested in our study and whether MIRU loci influence the expression of upstream and downstream gene through the mechanisms mentioned above still need further exploration.

## Author contributions

This research was designed by WYF, written by JC, and the data was analyzed by CXF.

### Conflict of interest statement

The authors declare that the research was conducted in the absence of any commercial or financial relationships that could be construed as a potential conflict of interest.

## Footnotes

^1^http://www.who.int/tb/publications/global_report/en/

^2^ http://www.miru-vntrplus.org/MIRU/files/MIRU-VNTRtypingmanualv6.pdf

^3^ http://www.ncbi.nlm.nih.gov/gene/885194

^4^http://combrex.bu.edu/DAI?command=SciBay&fun=gene&geneID=887734

^5^ http://www.ncbi.nlm.nih.gov/gene/888341

^6^http://www.uniprot.org/uniprot/I6Y4H2
