# Supplementary material for: Drug-resistant tuberculosis can be predicted by Mycobacterial interspersed repetitive unit locus
Source: Front Microbiol. 2015 Feb 24;6:147. doi: 10.3389/fmicb.2015.00147 (PMC4338821; doi:10.3389/fmicb.2015.00147)
Supplement: Supplementary file 1 [file Table1.DOCX]

Distribution and polymorphism of MIRU locus, and distribution of drug resistance rate

| Loci | Repeat | Frequency | % | *h*-value | INH resistance | | RFP resistance | | SM resistance | | EMB resistance | |
| --- | --- | --- | --- | --- | --- | --- | --- | --- | --- | --- | --- | --- |
|  |  |  |  |  | n | % | n | % | n | % | n | % |
| MIRU02 | 1 | 6 | 5.50 | 0.13 | 3 | 0.50 | 1 | 0.17 | 3 | 0.50 | 2 | 0.33 |
|  | 2 | 101 | 92.66 |  | 21 | 0.21 | 11 | 0.11 | 21 | 0.21 | 9 | 0.09 |
|  | 3 | 2 | 1.83 |  | 0 | 0.00 | 0 | 0.00 | 0 | 0.00 | 0 | 0.00 |
| MTUB04 | 1 | 12 | 11.01 | 0.68 | 0 | 0.00 | 1 | 0.08 | 0 | 0.00 | 0 | 0.00 |
|  | 2 | 54 | 49.54 |  | 7 | 0.13 | 2 | 0.04 | 5 | 0.09 | 3 | 0.06 |
|  | 3 | 15 | 13.76 |  | 4 | 0.27 | 2 | 0.13 | 5 | 0.33 | 1 | 0.07 |
|  | 4 | 22 | 20.18 |  | 11 | 0.50 | 6 | 0.27 | 13 | 0.59 | 7 | 0.32 |
|  | 5 | 6 | 5.50 |  | 2 | 0.33 | 1 | 0.17 | 1 | 0.17 | 0 | 0.00 |
| Etrc | 2 | 18 | 16.51 | 0.69 | 5 | 0.28 | 2 | 0.11 | 7 | 0.39 | 2 | 0.11 |
|  | 3 | 21 | 19.27 |  | 5 | 0.24 | 1 | 0.05 | 3 | 0.14 | 2 | 0.10 |
|  | 4 | 50 | 45.87 |  | 12 | 0.24 | 7 | 0.14 | 11 | 0.22 | 6 | 0.12 |
|  | 5 | 20 | 18.35 |  | 2 | 0.10 | 2 | 0.10 | 3 | 0.15 | 1 | 0.05 |
| MIRU04 | 1 | 4 | 3.67 | 0.30 | 0 | 0.00 | 0 | 0.00 | 0 | 0.00 | 0 | 0.00 |
|  | 2 | 90 | 82.57 |  | 24 | 0.27 | 12 | 0.13 | 24 | 0.27 | 11 | 0.12 |
|  | 3 | 5 | 4.59 |  | 0 | 0.00 | 0 | 0.00 | 0 | 0.00 | 0 | 0.00 |
|  | 4 | 3 | 2.75 |  | 0 | 0.00 | 0 | 0.00 | 0 | 0.00 | 0 | 0.00 |
|  | 5 | 6 | 5.50 |  | 0 | 0.00 | 0 | 0.00 | 0 | 0.00 | 0 | 0.00 |
|  | 6 | 1 | 0.92 |  | 0 | 0.00 | 0 | 0.00 | 0 | 0.00 | 0 | 0.00 |
| MIRU40 | 1 | 4 | 3.67 | 0.68 | 0 | 0.00 | 0 | 0.00 | 0 | 0.00 | 0 | 0.00 |
|  | 2 | 19 | 17.43 |  | 1 | 0.05 | 2 | 0.11 | 2 | 0.11 | 0 | 0.00 |
|  | 3 | 59 | 54.13 |  | 15 | 0.25 | 8 | 0.14 | 16 | 0.27 | 8 | 0.14 |
|  | 4 | 10 | 9.17 |  | 1 | 0.10 | 0 | 0.00 | 1 | 0.10 | 1 | 0.10 |
|  | 5 | 11 | 10.09 |  | 3 | 0.27 | 1 | 0.09 | 3 | 0.27 | 2 | 0.18 |
|  | 6 | 5 | 4.59 |  | 3 | 0.60 | 1 | 0.20 | 2 | 0.40 | 0 | 0.00 |
|  | 7 | 1 | 0.92 |  | 1 | 1.00 | 0 | 0.00 | 0 | 0.00 | 0 | 0.00 |
| MIRU10 | 2 | 5 | 4.59 | 0.66 | 1 | 0.20 | 0 | 0.00 | 1 | 0.20 | 0 | 0.00 |
|  | 3 | 55 | 50.46 |  | 16 | 0.29 | 8 | 0.15 | 16 | 0.29 | 8 | 0.15 |
|  | 4 | 24 | 22.02 |  | 4 | 0.17 | 2 | 0.08 | 3 | 0.13 | 2 | 0.08 |
|  | 5 | 18 | 16.51 |  | 3 | 0.17 | 2 | 0.11 | 2 | 0.11 | 1 | 0.06 |
|  | 6 | 3 | 2.75 |  | 0 | 0.00 | 0 | 0.00 | 2 | 0.67 | 0 | 0.00 |
|  | 7 | 1 | 0.92 |  | 0 | 0.00 | 0 | 0.00 | 0 | 0.00 | 0 | 0.00 |
|  | 9 | 2 | 1.83 |  | 0 | 0.00 | 0 | 0.00 | 0 | 0.00 | 0 | 0.00 |
|  | 10 | 1 | 0.92 |  | 0 | 0.00 | 0 | 0.00 | 0 | 0.00 | 0 | 0.00 |
| MIRU16 | 1 | 5 | 4.59 | 0.49 | 0 | 0.00 | 0 | 0.00 | 0 | 0.00 | 0 | 0.00 |
|  | 2 | 22 | 20.18 |  | 1 | 0.05 | 1 | 0.05 | 2 | 0.09 | 0 | 0.00 |
|  | 3 | 74 | 67.89 |  | 21 | 0.28 | 10 | 0.14 | 19 | 0.26 | 11 | 0.15 |
|  | 4 | 7 | 6.42 |  | 2 | 0.29 | 1 | 0.14 | 3 | 0.43 | 0 | 0.00 |
|  | 5 | 1 | 0.92 |  | 0 | 0.00 | 0 | 0.00 | 0 | 0.00 | 0 | 0.00 |
| MTUB21 | 1 | 5 | 4.59 | 0.66 | 1 | 0.20 | 1 | 0.20 | 0 | 0.00 | 1 | 0.20 |
|  | 2 | 6 | 5.5 |  | 0 | 0.00 | 0 | 0.00 | 1 | 0.17 | 0 | 0.00 |
|  | 3 | 57 | 52.29 |  | 8 | 0.14 | 5 | 0.09 | 7 | 0.12 | 3 | 0.05 |
|  | 4 | 22 | 20.18 |  | 5 | 0.23 | 1 | 0.05 | 5 | 0.23 | 1 | 0.05 |
|  | 5 | 14 | 12.84 |  | 9 | 0.64 | 5 | 0.36 | 10 | 0.71 | 6 | 0.43 |
|  | 6 | 4 | 3.67 |  | 1 | 0.25 | 0 | 0.00 | 1 | 0.25 | 0 | 0.00 |
|  | 10 | 1 | 0.92 |  | 0 | 0.00 | 0 | 0.00 | 0 | 0.00 | 0 | 0.00 |
| MIRU20 | 1 | 21 | 19.27 | 0.30 | 1 | 0.05 | 1 | 0.05 | 1 | 0.05 | 1 | 0.05 |
|  | 2 | 88 | 80.73 |  | 23 | 0.26 | 11 | 0.13 | 23 | 0.26 | 10 | 0.11 |
| OUB11b | 1 | 2 | 1.83 | 0.75 | 1 | 0.50 | 1 | 0.50 | 0 | 0.00 | 0 | 0.00 |
|  | 2 | 43 | 39.45 |  | 8 | 0.19 | 4 | 0.09 | 10 | 0.23 | 3 | 0.07 |
|  | 3 | 26 | 23.85 |  | 4 | 0.15 | 3 | 0.12 | 3 | 0.12 | 3 | 0.12 |
|  | 4 | 15 | 13.76 |  | 2 | 0.13 | 0 | 0.00 | 2 | 0.13 | 0 | 0.00 |
|  | 5 | 5 | 4.59 |  | 0 | 0.00 | 0 | 0.00 | 0 | 0.00 | 0 | 0.00 |
|  | 6 | 14 | 12.84 |  | 9 | 0.64 | 4 | 0.29 | 9 | 0.64 | 5 | 0.36 |
|  | 8 | 3 | 2.75 |  | 0 | 0.00 | 0 | 0.00 | 0 | 0.00 | 0 | 0.00 |
|  | 11 | 1 | 0.92 |  | 0 | 0.00 | 0 | 0.00 | 0 | 0.00 | 0 | 0.00 |
| Etra | 1 | 1 | 0.92 | 0.67 | 1 | 1.00 | 1 | 1.00 | 0 | 0.00 | 0 | 0.00 |
|  | 2 | 13 | 11.93 |  | 3 | 0.23 | 1 | 0.08 | 3 | 0.23 | 2 | 0.15 |
|  | 3 | 36 | 33.03 |  | 7 | 0.19 | 2 | 0.06 | 6 | 0.17 | 3 | 0.08 |
|  | 4 | 49 | 44.95 |  | 13 | 0.27 | 8 | 0.16 | 15 | 0.31 | 6 | 0.12 |
|  | 5 | 1 | 0.92 |  | 0 | 0.00 | 0 | 0.00 | 0 | 0.00 | 0 | 0.00 |
|  | 6 | 4 | 3.67 |  | 0 | 0.00 | 0 | 0.00 | 0 | 0.00 | 0 | 0.00 |
|  | 7 | 2 | 1.83 |  | 0 | 0.00 | 0 | 0.00 | 0 | 0.00 | 0 | 0.00 |
|  | 8 | 2 | 1.83 |  | 0 | 0.00 | 0 | 0.00 | 0 | 0.00 | 0 | 0.00 |
|  | 10 | 1 | 0.92 |  | 0 | 0.00 | 0 | 0.00 | 0 | 0.00 | 0 | 0.00 |
| MTUB29 | 2 | 3 | 2.75 | 0.24 | 0 | 0.00 | 0 | 0.00 | 0 | 0.00 | 0 | 0.00 |
|  | 3 | 11 | 10.09 |  | 1 | 0.09 | 1 | 0.09 | 1 | 0.09 | 1 | 0.09 |
|  | 4 | 94 | 86.24 |  | 23 | 0.24 | 11 | 0.12 | 23 | 0.24 | 10 | 0.11 |
|  | 5 | 1 | 0.92 |  | 0 | 0.00 | 0 | 0.00 | 0 | 0.00 | 0 | 0.00 |
| MTUB30 | 1 | 13 | 11.93 | 0.59 | 4 | 0.31 | 2 | 0.15 | 3 | 0.23 | 2 | 0.15 |
|  | 2 | 55 | 50.46 |  | 5 | 0.09 | 4 | 0.07 | 8 | 0.15 | 1 | 0.02 |
|  | 4 | 40 | 36.7 |  | 14 | 0.35 | 6 | 0.15 | 13 | 0.33 | 8 | 0.20 |
|  | 5 | 1 | 0.92 |  | 1 | 1.00 | 0 | 0.00 | 0 | 0.00 | 0 | 0.00 |
| Etrb | 1 | 18 | 16.51 | 0.37 | 2 | 0.11 | 1 | 0.06 | 2 | 0.11 | 1 | 0.06 |
|  | 2 | 84 | 77.06 |  | 22 | 0.26 | 11 | 0.13 | 22 | 0.26 | 10 | 0.12 |
|  | 4 | 4 | 3.67 |  | 0 | 0.00 | 0 | 0.00 | 0 | 0.00 | 0 | 0.00 |
|  | 5 | 2 | 1.83 |  | 0 | 0.00 | 0 | 0.00 | 0 | 0.00 | 0 | 0.00 |
|  | 6 | 1 | 0.92 |  | 0 | 0.00 | 0 | 0.00 | 0 | 0.00 | 0 | 0.00 |
| MIRU23 | 2 | 1 | 0.92 | 0.38 | 0 | 0.00 | 0 | 0.00 | 0 | 0.00 | 0 | 0.00 |
|  | 3 | 2 | 1.83 |  | 0 | 0.00 | 0 | 0.00 | 0 | 0.00 | 0 | 0.00 |
|  | 4 | 2 | 1.83 |  | 0 | 0.00 | 0 | 0.00 | 1 | 0.50 | 0 | 0.00 |
|  | 5 | 83 | 76.15 |  | 21 | 0.25 | 10 | 0.12 | 22 | 0.27 | 11 | 0.13 |
|  | 6 | 18 | 16.51 |  | 2 | 0.11 | 1 | 0.06 | 1 | 0.06 | 0 | 0.00 |
|  | 7 | 3 | 2.75 |  | 1 | 0.33 | 1 | 0.33 | 0 | 0.00 | 0 | 0.00 |
| MIRU24 | 1 | 100 | 91.74 | 0.14 | 24 | 0.24 | 12 | 0.12 | 24 | 0.24 | 11 | 0.11 |
|  | 2 | 9 | 8.26 |  | 0.00 | 0.00 | 0 | 0.00 | 0 | 0.00 | 0 | 0.00 |
| MIRU26 | 1 | 8 | 7.34 | 0.71 | 0 | 0.00 | 0 | 0.00 | 0 | 0.00 | 0 | 0.00 |
|  | 2 | 10 | 9.17 |  | 0 | 0.00 | 0 | 0.00 | 0 | 0.00 | 0 | 0.00 |
|  | 3 | 2 | 1.83 |  | 0 | 0.00 | 0 | 0.00 | 0 | 0.00 | 0 | 0.00 |
|  | 4 | 30 | 27.52 |  | 8 | 0.27 | 3 | 0.10 | 7 | 0.23 | 3 | 0.10 |
|  | 5 | 48 | 44.04 |  | 12 | 0.25 | 7 | 0.15 | 11 | 0.23 | 7 | 0.15 |
|  | 6 | 1 | 0.92 |  | 0 | 0.00 | 0 | 0.00 | 0 | 0.00 | 0 | 0.00 |
|  | 7 | 8 | 7.34 |  | 4 | 0.50 | 2 | 0.25 | 4 | 0.50 | 1 | 0.13 |
|  | 8 | 1 | 0.92 |  | 0 | 0.00 | 0 | 0.00 | 1 | 1.00 | 0 | 0.00 |
|  | 9 | 1 | 0.92 |  | 0 | 0.00 | 0 | 0.00 | 1 | 1.00 | 0 | 0.00 |
| MIRU27 | 1 | 3 | 2.75 | 0.10 | 1 | 0.33 | 1 | 0.33 | 0 | 0.00 | 1 | 0.33 |
|  | 3 | 103 | 94.5 |  | 23 | 0.22 | 11 | 0.11 | 24 | 0.23 | 10 | 0.10 |
|  | 4 | 3 | 2.75 |  | 0 | 0.00 | 0 | 0.00 | 0 | 0.00 | 0 | 0.00 |
| MTUB34 | 1 | 2 | 1.83 | 0.29 | 0 | 0.00 | 0 | 0.00 | 0 | 0.00 | 0 | 0.00 |
|  | 2 | 14 | 12.84 |  | 6 | 0.43 | 1 | 0.07 | 5 | 0.36 | 1 | 0.07 |
|  | 3 | 90 | 82.57 |  | 18 | 0.20 | 11 | 0.12 | 18 | 0.20 | 10 | 0.11 |
|  | 4 | 1 | 0.92 |  | 0 | 0.00 | 0 | 0.00 | 1 | 1.00 | 0 | 0.00 |
|  | 5 | 2 | 1.83 |  | 0 | 0.00 | 0 | 0.00 | 0 | 0.00 | 0 | 0.00 |
| MIRU31 | 2 | 10 | 9.17 | 0.67 | 4 | 0.40 | 3 | 0.30 | 3 | 0.30 | 2 | 0.20 |
|  | 3 | 53 | 48.62 |  | 5 | 0.09 | 1 | 0.02 | 3 | 0.06 | 2 | 0.04 |
|  | 4 | 21 | 19.27 |  | 3 | 0.14 | 2 | 0.10 | 5 | 0.24 | 1 | 0.05 |
|  | 5 | 23 | 21.1 |  | 12 | 0.52 | 6 | 0.26 | 13 | 0.57 | 6 | 0.26 |
|  | 6 | 2 | 1.83 |  | 0 | 0.00 | 0 | 0.00 | 0 | 0.00 | 0 | 0.00 |
| MTUB39 | 1 | 2 | 1.83 | 0.67 | 0 | 0.00 | 0 | 0.00 | 0 | 0.00 | 0 | 0.00 |
|  | 2 | 22 | 20.18 |  | 7 | 0.32 | 5 | 0.23 | 5 | 0.23 | 2 | 0.09 |
|  | 3 | 57 | 52.29 |  | 17 | 0.30 | 7 | 0.12 | 19 | 0.33 | 9 | 0.16 |
|  | 4 | 12 | 11.01 |  | 0 | 0.00 | 0 | 0.00 | 0 | 0.00 | 0 | 0.00 |
|  | 5 | 2 | 1.83 |  | 0 | 0.00 | 0 | 0.00 | 0 | 0.00 | 0 | 0.00 |
|  | 6 | 5 | 4.59 |  | 0 | 0.00 | 0 | 0.00 | 0 | 0.00 | 0 | 0.00 |
|  | 7 | 2 | 1.83 |  | 0 | 0.00 | 0 | 0.00 | 0 | 0.00 | 0 | 0.00 |
|  | 8 | 2 | 1.83 |  | 0 | 0.00 | 0 | 0.00 | 0 | 0.00 | 0 | 0.00 |
|  | 9 | 2 | 1.83 |  | 0 | 0.00 | 0 | 0.00 | 0 | 0.00 | 0 | 0.00 |
|  | 10 | 2 | 1.83 |  | 0 | 0.00 | 0 | 0.00 | 0 | 0.00 | 0 | 0.00 |
|  | 13 | 1 | 0.92 |  | 0 | 0.00 | 0 | 0.00 | 0 | 0.00 | 0 | 0.00 |
| OUB26 | 2 | 1 | 0.92 | 0.81 | 0 | 0.00 | 0 | 0.00 | 1 | 1.00 | 0 | 0.00 |
|  | 3 | 4 | 3.67 |  | 0 | 0.00 | 0 | 0.00 | 0 | 0.00 | 0 | 0.00 |
|  | 4 | 12 | 11.01 |  | 0 | 0.00 | 0 | 0.00 | 0 | 0.00 | 0 | 0.00 |
|  | 5 | 17 | 15.6 |  | 1 | 0.06 | 0 | 0.00 | 1 | 0.06 | 0 | 0.00 |
|  | 6 | 31 | 28.44 |  | 9 | 0.29 | 4 | 0.13 | 7 | 0.23 | 4 | 0.13 |
|  | 7 | 21 | 19.27 |  | 5 | 0.24 | 4 | 0.19 | 5 | 0.24 | 3 | 0.14 |
|  | 8 | 21 | 19.27 |  | 9 | 0.43 | 4 | 0.19 | 10 | 0.48 | 4 | 0.19 |
|  | 9 | 1 | 0.92 |  | 0 | 0.00 | 0 | 0.00 | 0 | 0.00 | 0 | 0.00 |
|  | 10 | 1 | 0.92 |  | 0 | 0.00 | 0 | 0.00 | 0 | 0.00 | 0 | 0.00 |
| OUB4156 | 0 | 2 | 1.83 | 0.65 | 0 | 0.00 | 0 | 0.00 | 0 | 0.00 | 0 | 0.00 |
|  | 1 | 20 | 18.35 |  | 4 | 0.20 | 0 | 0.00 | 3 | 0.15 | 1 | 0.05 |
|  | 2 | 56 | 51.38 |  | 17 | 0.30 | 10 | 0.18 | 18 | 0.32 | 9 | 0.16 |
|  | 3 | 22 | 20.18 |  | 1 | 0.05 | 1 | 0.05 | 0 | 0.00 | 1 | 0.05 |
|  | 4 | 9 | 8.26 |  | 2 | 0.22 | 1 | 0.11 | 3 | 0.33 | 0 | 0.00 |
| MIRU39 | 1 | 1 | 0.92 | 0.44 | 0 | 0.00 | 0 | 0.00 | 0 | 0.00 | 0 | 0.00 |
|  | 2 | 75 | 68.81 |  | 10 | 0.13 | 7 | 0.09 | 8 | 0.11 | 4 | 0.05 |
|  | 3 | 31 | 28.44 |  | 13 | 0.42 | 4 | 0.13 | 15 | 0.48 | 6 | 0.19 |
|  | 4 | 2 | 1.83 |  | 1 | 0.50 | 1 | 0.50 | 1 | 0.50 | 1 | 0.50 |

Univariate analysis of influencing factors of four anti-tuberculosis drug resistance

| Model | Loci | B | S.E. | Wald*χ^2^* | *P*-value | OR | OR95% CI | |
| --- | --- | --- | --- | --- | --- | --- | --- | --- |
|  |  |  |  |  |  |  | Lower | Upper |
| INH | MIRU02 | -1.42 | 0.82 | 3.07 | 0.08 | 0.24 | 0.05 | 1.19 |
|  | MTUB04 | 0.79 | 0.23 | 12.27 | 0.00 | 2.20 | 1.41 | 3.41 |
|  | Etrc | -0.28 | 0.24 | 1.35 | 0.25 | 0.76 | 0.48 | 1.21 |
|  | MIRU04 | -0.77 | 0.50 | 2.37 | 0.12 | 0.46 | 0.17 | 1.24 |
|  | MIRU40 | 0.52 | 0.20 | 6.89 | 0.01 | 1.68 | 1.14 | 2.48 |
|  | MIRU10 | -0.44 | 0.26 | 2.96 | 0.09 | 0.64 | 0.39 | 1.06 |
|  | MIRU16 | 0.84 | 0.41 | 4.16 | 0.04 | 2.31 | 1.03 | 5.14 |
|  | MTUB21 | 0.42 | 0.20 | 4.55 | 0.03 | 1.52 | 1.03 | 2.23 |
|  | MIRU20 | 1.96 | 1.05 | 3.45 | 0.06 | 7.08 | 0.90 | 55.74 |
|  | OUB11b | 0.15 | 0.12 | 1.53 | 0.22 | 1.17 | 0.92 | 1.48 |
|  | Etra | -0.32 | 0.22 | 1.98 | 0.16 | 0.73 | 0.47 | 1.13 |
|  | MTUB29 | -0.32 | 0.22 | 1.98 | 0.16 | 0.73 | 0.47 | 1.13 |
|  | MTUB30 | 0.50 | 0.21 | 5.44 | 0.02 | 1.64 | 1.08 | 2.49 |
|  | Etrb | -0.19 | 0.33 | 0.34 | 0.56 | 0.83 | 0.43 | 1.57 |
|  | MIRU23 | 0.09 | 0.36 | 0.06 | 0.81 | 1.09 | 0.54 | 2.20 |
|  | MIRU24 | -20.05 | 1340.00 | 0.00 | 1.00 | 0.00 | 0.00 | .- |
|  | MIRU26 | 0.39 | 0.17 | 5.12 | 0.02 | 1.47 | 1.05 | 2.06 |
|  | MIRU27 | -0.46 | 0.56 | 0.67 | 0.41 | 0.63 | 0.21 | 1.89 |
|  | MTUB34 | -0.62 | 0.45 | 1.91 | 0.17 | 0.54 | 0.22 | 1.30 |
|  | MIRU31 | 0.50 | 0.24 | 4.45 | 0.04 | 1.65 | 1.04 | 2.62 |
|  | MTUB39 | -0.64 | 0.29 | 5.03 | 0.03 | 0.53 | 0.30 | 0.92 |
|  | OUB26 | 0.52 | 0.19 | 7.94 | 0.01 | 1.69 | 1.17 | 2.43 |
|  | OUB4156 | -0.18 | 0.27 | 0.44 | 0.51 | 0.84 | 0.49 | 1.42 |
|  | MIRU39 | 1.39 | 0.44 | 9.80 | 0.00 | 4.02 | 1.68 | 9.60 |
| RFP | MIRU02 | -0.65 | 1.02 | 0.41 | 0.52 | 0.52 | 0.07 | 3.86 |
|  | MTUB04 | 0.65 | 0.28 | 5.54 | 0.02 | 1.91 | 1.11 | 3.28 |
|  | Etrc | 0.11 | 0.33 | 0.12 | 0.73 | 1.12 | 0.59 | 2.11 |
|  | MIRU04 | -0.69 | 0.65 | 1.12 | 0.29 | 0.50 | 0.14 | 1.80 |
|  | MIRU40 | 0.03 | 0.26 | 0.01 | 0.92 | 1.03 | 0.61 | 1.72 |
|  | MIRU10 | -0.24 | 0.30 | 0.66 | 0.42 | 0.79 | 0.44 | 1.41 |
|  | MIRU16 | 0.58 | 0.49 | 1.36 | 0.24 | 1.78 | 0.68 | 4.69 |
|  | MTUB21 | 0.18 | 0.22 | 0.62 | 0.43 | 1.19 | 0.77 | 1.85 |
|  | MIRU20 | 1.05 | 1.07 | 0.96 | 0.33 | 2.86 | 0.35 | 23.46 |
|  | OUB11b | 0.04 | 0.17 | 0.05 | 0.83 | 1.04 | 0.75 | 1.44 |
|  | Etra | -0.20 | 0.28 | 0.50 | 0.48 | 0.82 | 0.48 | 1.42 |
|  | MTUB29 | 0.43 | 0.84 | 0.27 | 0.60 | 1.54 | 0.30 | 7.92 |
|  | MTUB30 | 0.17 | 0.27 | 0.39 | 0.53 | 1.18 | 0.70 | 2.01 |
|  | Etrb | -0.17 | 0.44 | 0.15 | 0.70 | 0.84 | 0.36 | 2.00 |
|  | MIRU23 | 0.31 | 0.48 | 0.40 | 0.53 | 1.36 | 0.53 | 3.51 |
|  | MIRU24 | -19.21 | 1340.00 | 0.00 | 1.00 | 0.00 | 0.00 |  |
|  | MIRU26 | 0.37 | 0.21 | 2.99 | 0.08 | 1.45 | 0.95 | 2.20 |
|  | MIRU27 | -0.78 | 0.60 | 1.69 | 0.19 | 0.46 | 0.14 | 1.49 |
|  | MTUB34 | 0.15 | 0.59 | 0.07 | 0.80 | 1.16 | 0.37 | 3.69 |
|  | MIRU31 | 0.38 | 0.30 | 1.57 | 0.21 | 1.46 | 0.81 | 2.65 |
|  | MTUB39 | -0.78 | 0.41 | 3.58 | 0.06 | 0.46 | 0.20 | 1.03 |
|  | OUB26 | 0.50 | 0.24 | 4.36 | 0.04 | 1.65 | 1.03 | 2.64 |
|  | OUB4156 | 0.15 | 0.35 | 0.19 | 0.67 | 1.16 | 0.59 | 2.28 |
|  | MIRU39 | 0.70 | 0.54 | 1.71 | 0.19 | 2.01 | 0.71 | 5.73 |
| SM | MIRU02 | -1.43 | 0.82 | 3.07 | 0.08 | 0.24 | 0.05 | 1.19 |
|  | MTUB04 | 0.89 | 0.23 | 14.71 | 0.00 | 2.44 | 1.55 | 3.84 |
|  | Etrc | -0.33 | 0.24 | 1.95 | 0.16 | 0.72 | 0.45 | 1.14 |
|  | MIRU04 | -0.77 | 0.50 | 2.37 | 0.12 | 0.46 | 0.17 | 1.24 |
|  | MIRU40 | 0.22 | 0.19 | 1.29 | 0.26 | 1.25 | 0.85 | 1.82 |
|  | MIRU10 | -0.27 | 0.22 | 1.48 | 0.22 | 0.76 | 0.49 | 1.18 |
|  | MIRU16 | 0.84 | 0.41 | 4.16 | 0.04 | 2.31 | 1.03 | 5.14 |
|  | MTUB21 | 0.54 | 0.21 | 6.58 | 0.01 | 1.72 | 1.14 | 2.61 |
|  | MIRU20 | 1.96 | 1.05 | 3.45 | 0.06 | 7.08 | 0.90 | 55.74 |
|  | OUB11b | 0.15 | 0.12 | 1.53 | 0.22 | 1.17 | 0.92 | 1.48 |
|  | Etra | -0.14 | 0.20 | 0.51 | 0.48 | 0.87 | 0.59 | 1.28 |
|  | MTUB29 | 0.95 | 0.76 | 1.57 | 0.21 | 2.58 | 0.59 | 11.38 |
|  | MTUB30 | 0.32 | 0.21 | 2.40 | 0.12 | 1.38 | 0.92 | 2.07 |
|  | Etrb | -0.19 | 0.33 | 0.34 | 0.56 | 0.83 | 0.43 | 1.57 |
|  | MIRU23 | -0.40 | 0.35 | 1.31 | 0.25 | 0.67 | 0.34 | 1.33 |
|  | MIRU24 | -20.05 | 1339.65 | 0.00 | 1.00 | 0.00 | 0.00 |  |
|  | MIRU26 | 0.67 | 0.21 | 10.33 | 0.00 | 1.95 | 1.30 | 2.92 |
|  | MIRU27 | 0.29 | 0.70 | 0.17 | 0.68 | 1.34 | 0.34 | 5.30 |
|  | MTUB34 | -0.22 | 0.44 | 0.26 | 0.61 | 0.80 | 0.34 | 1.91 |
|  | MIRU31 | 0.79 | 0.25 | 10.04 | 0.00 | 2.21 | 1.35 | 3.61 |
|  | MTUB39 | -0.50 | 0.25 | 4.00 | 0.05 | 0.61 | 0.37 | 0.99 |
|  | OUB26 | 0.46 | 0.18 | 6.49 | 0.01 | 1.58 | 1.11 | 2.25 |
|  | OUB4156 | -0.04 | 0.27 | 0.02 | 0.89 | 0.96 | 0.57 | 1.62 |
|  | MIRU39 | 1.81 | 0.47 | 14.71 | 0.00 | 6.11 | 2.42 | 15.40 |
| EMB | MIRU02 | -1.67 | 0.91 | 3.34 | 0.07 | 0.19 | 0.03 | 1.13 |
|  | MTUB04 | 0.67 | 0.29 | 5.41 | 0.02 | 1.95 | 1.11 | 3.41 |
|  | Etrc | -0.14 | 0.33 | 0.18 | 0.68 | 0.87 | 0.46 | 1.65 |
|  | MIRU04 | -0.68 | 0.68 | 1.02 | 0.31 | 0.50 | 0.13 | 1.90 |
|  | MIRU40 | 0.18 | 0.26 | 0.50 | 0.48 | 1.20 | 0.72 | 1.99 |
|  | MIRU10 | -0.41 | 0.36 | 1.29 | 0.26 | 0.67 | 0.33 | 1.34 |
|  | MIRU16 | 0.57 | 0.51 | 1.24 | 0.27 | 1.77 | 0.65 | 4.82 |
|  | MTUB21 | 0.31 | 0.22 | 2.00 | 0.16 | 1.37 | 0.89 | 2.10 |
|  | MIRU20 | 0.94 | 1.08 | 0.76 | 0.38 | 2.56 | 0.31 | 21.22 |
|  | OUB11b | 0.21 | 0.16 | 1.84 | 0.18 | 1.23 | 0.91 | 1.67 |
|  | Etra | -0.25 | 0.30 | 0.67 | 0.41 | 0.78 | 0.44 | 1.41 |
|  | MTUB29 | 0.37 | 0.85 | 0.19 | 0.66 | 1.45 | 0.28 | 7.56 |
|  | MTUB30 | 0.57 | 0.30 | 3.56 | 0.06 | 1.76 | 0.98 | 3.17 |
|  | Etrb | -0.19 | 0.46 | 0.16 | 0.69 | 0.83 | 0.33 | 2.06 |
|  | MIRU23 | -0.33 | 0.45 | 0.54 | 0.46 | 0.72 | 0.30 | 1.73 |
|  | MIRU24 | -19.11 | 1339.66 | 0.00 | 1.00 | 0.00 | 0.00 |  |
|  | MIRU26 | 0.27 | 0.22 | 1.61 | 0.20 | 1.31 | 0.86 | 2.00 |
|  | MIRU27 | -0.83 | 0.61 | 1.86 | 0.17 | 0.44 | 0.13 | 1.44 |
|  | MTUB34 | 0.12 | 0.61 | 0.04 | 0.85 | 1.12 | 0.34 | 3.74 |
|  | MIRU31 | 0.47 | 0.32 | 2.18 | 0.14 | 1.60 | 0.86 | 2.96 |
|  | MTUB39 | -0.41 | 0.32 | 1.59 | 0.21 | 0.67 | 0.35 | 1.25 |
|  | OUB26 | 0.49 | 0.25 | 3.98 | 0.05 | 1.64 | 1.01 | 2.66 |
|  | OUB4156 | -0.22 | 0.37 | 0.34 | 0.56 | 0.80 | 0.39 | 1.67 |
|  | MIRU39 | 1.45 | 0.57 | 6.60 | 0.01 | 4.27 | 1.41 | 12.92 |
